# Impact of growth trajectory on sexual maturation in layer chickens

**DOI:** 10.3389/fphys.2023.1174238

**Published:** 2023-05-05

**Authors:** Mohammad A. Bahry, Charlene Hanlon, Clara J. Ziezold, Sierra Schaus, Grégoy Y. Bédécarrats

**Affiliations:** ^1^ Department of Animal Biosciences, University of Guelph, Guelph, ON, Canada; ^2^ Department of Poultry Science, College of Agriculture, Auburn University, Auburn, AL, United States

**Keywords:** layers, body weight, body composition, sexual maturation, egg production

## Abstract

Recent studies showed that apart from photostimulation, metabolic triggers may independently activate sexual maturation and egg production in chickens. However, the origin, mode of action, and specific target(s) of this metabolic control remain unknown. Beyond body weight (BW), we hypothesize that body composition (BC) and associated specific metabolic signals are involved. Thus, this study was conducted to determine the BW and BC thresholds triggering spontaneous sexual maturation in layer pullets under different growth trajectories. Day-old Lohman LSL lite and Lohman brown lite chicks (*n* = 210 each) raised in brooding cages under *ad libitum* (AL) feeding until 8 weeks of age were randomly allocated into individual cages and assigned to one of 3 experimental growth profiles; AL, breeder’s target (T), restricted 20% below target (R), (*n* = 70 birds/profile/strain). Birds had free access to water throughout the trial. All hens were maintained on 10 h of light (10 lux) throughout the rest of the study. Blood and tissue samples were collected throughout the study to measure plasma estradiol (E_2_) concentrations and organ weights, respectively. Furthermore, carcasses were subjected to Dual-energy X-ray absorptiometry (DEXA) analyses. All analyses were completed with SAS using the MIXED procedure. Results show that R treatment slowed (*p* < 0.001) growth, delayed age at first egg (FE) and egg production (*p* < 0.001) and resulted in lower BW at FE (*p* < 0.001), lower ovary weight and number of follicles (*p* < 0.001) compared to AL in both strains, whereas, the strain significantly impacted body weight (*p* < 0.0001), ovary weight (*p* < 0.001), BW at FE (*p* < 0.001), age at FE (*p* < 0.001), egg production (*p* < 0.0001), E_2_ (*p* < 0.0001) and body composition (*p* < 0.05). For DEXA, AL feeding (*p* < 0.001) increased fat deposition compared to R. Furthermore, there was a positive correlation between plasma E_2_ and bone mineral content (*p* < 0.01) and bone mineral density (*p* < 0.01). In conclusion, feed allocation impacted growth and BC in a strain dependent manner which resulted in differing age at sexual maturation and egg production. Furthermore, a body fat threshold between 10% to 15% appears to be required for the occurrence of spontaneously sexual maturation in laying hens.

## 1 Introduction

Chickens are seasonal breeders, relying on changes in photoperiod to initiate and terminate reproduction. However, it has also been shown that apart from photostimulation, metabolic triggers can independently activate sexual maturation and egg production in both layers and broiler breeders (van der Klein et al., 2018a; Baxter and Bédécarrats, 2019; Hanlon et al., 2021). Thus, it is evident that growth trajectories can influence the initiation of reproduction, possibly at all levels of the hypothalamic-pituitary-ovarian axis. However, the origin, mode of action, and specific target(s) of this metabolic control remain to be determined. Beyond body weight (**BW**), body composition (**BC**) may be directly related to metabolic input on sexual maturation in chickens. Studies in broiler breeders and quail revealed that specific BW and BC are required to initiate maturation (Bornstein et al., 1984; Yang et al., 2013). Altering growth trajectories (van Emous et al., 2013), feed allocation (Robinson et al., 2007), or dietary composition (Spratt and Leeson, 1987) have all been proven management strategies that can alter BC. Specifically, Heijmans et al. (2021) reported that birds on a 15% elevated growth curve demonstrated greater abdominal fat accumulation. Interestingly, van der Klein et al. (2018a) showed that a fat pad of 2.5% of BW or higher was required for broiler breeder hens to enter lay, as hens with a lower fat pad (1.5%) did not enter lay before 55 weeks of age (**woa**). Since the abdominal fat pad was proposed as an accurate indicator of overall fat accumulations in chickens (Sato et al., 2009), signalling from adipose tissue is likely behind the metabolic threshold involved in initiating sexual maturation. Therefore, achieving a critical BC threshold during the juvenile stage may be required to support egg formation throughout a laying cycle (Hanlon et al., 2020). Thus, by controlling for BW and hence BC, the first aim of this study was to determine the carcass fat and lean thresholds associated with spontaneous sexual maturation in brown **(B**) and white (**W**) strains of modern commercial layers.

The hypothalamic-pituitary-gonadal (HPG) axis controls reproduction in chickens through a tight balance between stimulatory (chicken gonadotropin-releasing hormone I—cGnRH-I) and inhibitory (gonadotropin inhibitory hormone—GnIH) inputs (Bédécarrats, 2015; Hanlon et al., 2020). In addition to its reproductive function, GnIH also possesses orexigenic effects that stimulate feed intake in chickens (Tachibana et al., 2005; Chowdhury et al., 2012). Increased levels of GnIH directly inhibit cGnRH-I (Bentley et al., 2003; Bentley et al., 2008) within the hypothalamus and reduce the release of gonadotropins (luteinizing hormone; LH and follicle-stimulating hormone; FSH; Tsutsui et al., 2000; Ciccone et al., 2004; Ikemoto and Park, 2005; Ubuka et al., 2006) by the anterior pituitary gland. Once released into the systemic circulation, LH and FSH initiate the maturation of small white follicles (**SWF**s), which gradually increase their production of estradiol (E_2_; Williams and Sharp, 1978; Robinson and Etches, 1986). These SWFs will continue to progress and develop, undergo cyclic recruitment into the hierarchy (F1-F6; Johnson, 1993), and eventually result in the daily ovulation of the largest follicle (F1) (Johnson et al., 1996). In broiler breeders, the number of hierarchical follicles increases with elevations in BW under *ad libitum* feeding (Hocking, 1993), resulting in a double hierarchy which can be controlled through feed restriction (Hocking et al., 1989). However, the link between the feeding paradigm and follicular maturation remains unclear in laying hens. Non-etheless, we hypothesize that metabolic status will impact the timing of ovarian maturation. Thus, the second aim of this study was to investigate whether induced changes in BW and composition can alter ovarian follicular development and maturation, and therefore E_2_ profiles.

Additionally, during sexual maturation, skeletal development shifts from longitudinal growth to the medullary formation (Miller and Bowman, 1981; Dacke et al., 1993; Whitehead and Fleming, 2000), providing a mineral reservoir to supplement dietary calcium (**Ca**) for eggshell formation (Mueller et al., 1964; Miller, 1977). This occurs concurrently with the rise in E_2_, and studies in quail showed that the development of medullary bone and an elevation in total plasma Ca is triggered 72–120 h following estradiol valerate administration (Miller and Bowman, 1981). The authors said that the changes in plasma E_2_ concentration at the onset of sexual maturation affect bone physiology in high-producing quail to provide enough Ca required in the egg-production process. Eusemann et al. (2020) reported that bone mineral density (**BMD**) was higher in non-laying hens compared to laying hens. Furthermore, exogenous E_2_ administration increased BMD and reduced the risk of fracture in non-laying hens, while it increased the risk of fracture in laying hens. It was found that the radiographic bone density was higher in traditional breeds with lower egg production compared to the high-producing birds (Hocking et al., 2003). This was supported by Habig et al. (2017), who reported that hens with higher laying performance showed a lower BMD than those with moderate laying performance. Thus, the final aim of this study was to investigate the impact of strain and differing growth curves on laying status, plasma E_2_, bone mineral content (**BMC**) and BMD.

## 2 Materials and methods

### 2.1 Animals and experimental design

Day-old Lohmann LSL lite (**W**) and Lohmann brown lite (**B**) *Gallus gallus domesticus* (*n* = 210 each) were purchased from a local hatchery (Archers Hatchery, Brighton, ON) and housed at the Arkell Poultry Research Station (University of Guelph, Guelph, ON) in colony brooding cages (76 cm wide × 66 cm deep × 40 cm tall; *n* = 7 cages; 30 chicks/cage) for the first 4 woa, then density was reduced to 14 chicks/cage (*n* = 15 cages) until 8 woa. Birds were fed *ad libitum* and maintained under the photo schedule according to the breeder’s management guide for North America (Table 1). At 8 woa, pullets were randomly allocated into individual cages across two rooms (*n* = 210 cages/room) and assigned to one of 3 experimental growth profiles, *ad libitum* (**AL**), breeder’s recommended target (**T**), and feed restricted to achieve a BW 20% below target (**R**) (*n* = 70 birds/profile/strain). From 8 woa, individual BWs were recorded weekly and feed allocation for T and R birds was determined individually based on actual BWs ( $\frac{Target\;bodyweight\times Target\;feed\;intake}{Actual\;bodyweight}$ ). All hens were fed the same commercial diet (Floradale Feed Mill Limited, Floradale, Ontario, Canada) formulated to meet or exceed the NRC requirements (National Research Council, 1994), including a starter crumble diet (0–6 woa with 21% energy), pullet grower crumble diet (7–16 woa with 18% energy), and layer breeder (17 woa to end of the trial). All hens were maintained on a 10 h photoperiod (10 lux) throughout the rest of the study to avoid confounding photostimulatory with metabolic triggers. This experiment was approved by the Animal Care Committee at the University of Guelph, and all procedures and management followed the guidelines from the Canadian Council for Animal Care (CCAC, 2009).

**TABLE 1:** Lighting schedule (no photostimulation).

Program start (day)	Photoperiod (h)	Intensity (lux)
0	16	40
4	16	35
7	14	30
12	13	25
19	12	20
26	11	15
33 to end (25 woa)	10	10

woa, weeks of age.

### 2.2 Growth and production performance

The growth trajectory was determined by calculating weekly weight gain. Individual egg production was recorded daily and expressed as a weekly hen-day production percentage. Furthermore, BW and age at first egg (**AFE**) were recorded to determine the timing of sexual maturation for each hen. At 14, 16, 18, 20, 22, and 25 woa, 6 birds per treatment and strain were weighed and approximately 3 mL of blood was collected from the brachial vein and placed in sodium heparin tubes for later processing. Birds were then euthanized *via* cervical dislocation. Ovaries were weighed without large yellow follicles (**LYF**s; follicles larger than 8 mm) and the total number of SWFs and LYFs from each ovary were counted. Ovary weight was expressed relative to BW.

### 2.3 Body composition

After removing the head and ovary, BC was analyzed from the same carcasses at 14, 16, 18, 20, 22, and 25 woa (*n* = 6 birds/treatment/strain) *via* Dual Energy X-ray Absorptiometry (DXA). After euthanasia, carcasses were placed at 4°C to slowly decrease the core temperature before being stored at −20°C until analysis. Before DXA scanning, all carcasses were placed at 4°C for 48 h and then at room temperature until thawed. Scans were performed using a Lunar Prodigy Advance DXA scanner (GE Healthcare, Madison, WI) with the enCore software version 16. A quality assurance block was conducted daily prior to the scans to calibrate the machine. Carcasses were placed on the scanning bed in a uniform lateral position. DXA scans were run using the small animal setting in small mode according to Swennen et al. (2004) to measure, BMC (g), total bone area (cm^2^), fat tissue weight (g), lean tissue weight (g) and total tissue weight (g). Fat % and lean % were calculated as $\left(\frac{fat\;or\;lean\;weight}{Tissue\;weight}\right)\times 100$ and BMD (g/cm^2^) was calculated as $\left(\frac{BMC}{total\;bone\;area}\right)\times 100$ .

### 2.4 Estradiol (E2) analysis

In addition to the blood sample collected from sacrificed birds described in Section 2.2, repeated blood samples were also collected from focal birds (*n* = 10 birds/treatment/strain) every 2 weeks from 10–14 woa and weekly from 14 to 25 woa. Plasma was recovered by centrifugation (Centrifuge J6-MI, Beckman Coulter, Inc., United States) for 15 min at 900 × g at 4°C and stored at −20°C until extractions and assays were completed as described by Hanlon et al. (2021). Briefly, plasma samples underwent a fat extraction using the cold ethanol extraction protocol described by Baxter et al. (2014). The DetectX commercial estradiol ELISA was used to measure E_2_ concentration following the manufacturer’s protocol (DetectX 17β-estradiol enzyme immunoassay #K030-H5, Arbor Assays, Ann Arbor, Michigan). Samples were assayed in duplicates, with optical densities measured at 450 nm using a microplate spectrophotometer (Model 550, Bio-Rad, CA, United States). Data were analyzed using the MyAssays software (www.myassays.com/arbor-assays-estradiol-eia-kit.assay) with a four-parameter logistic curve and the intra-assay and inter-assay coefficient of variance (CV) were <15%.

### 2.5 Statistical analyses

All statistical analyses were performed using SAS v9.4 (SAS Institute, Cary, NC). BW and egg production parameters, as well as E_2_ concentrations from focal birds, were analyzed by a three-way ANOVA (PROC MIXED) with respect to strain, treatment, age, and their interactions. All other parameters, including measures of BC, E_2_ concentrations from sacrificed birds, relative ovary weight, and the number of follicles were analyzed using a two-way ANOVA (PROC MIXED), whereby strain, treatment and their interactions were independent variables. Random effects for all measures included room, tier, and their interaction. Means were separated by the least squares means of fixed effects (LSMEANS), the test of least significant differences (PDIFF) and Tukey’s multiple comparisons. When appropriate, repeated measures on individual hens over time were included in the model with respect to hen ID. Pearson correlations (PROC CORR) were performed within treatments across variables, including, E_2_, BMC and BMD. Statements of significance were based on *p* < 0.05 and data were expressed as mean ± SEM.

## 3 Results

### 3.1 Body weight

**FIGURE 1:**
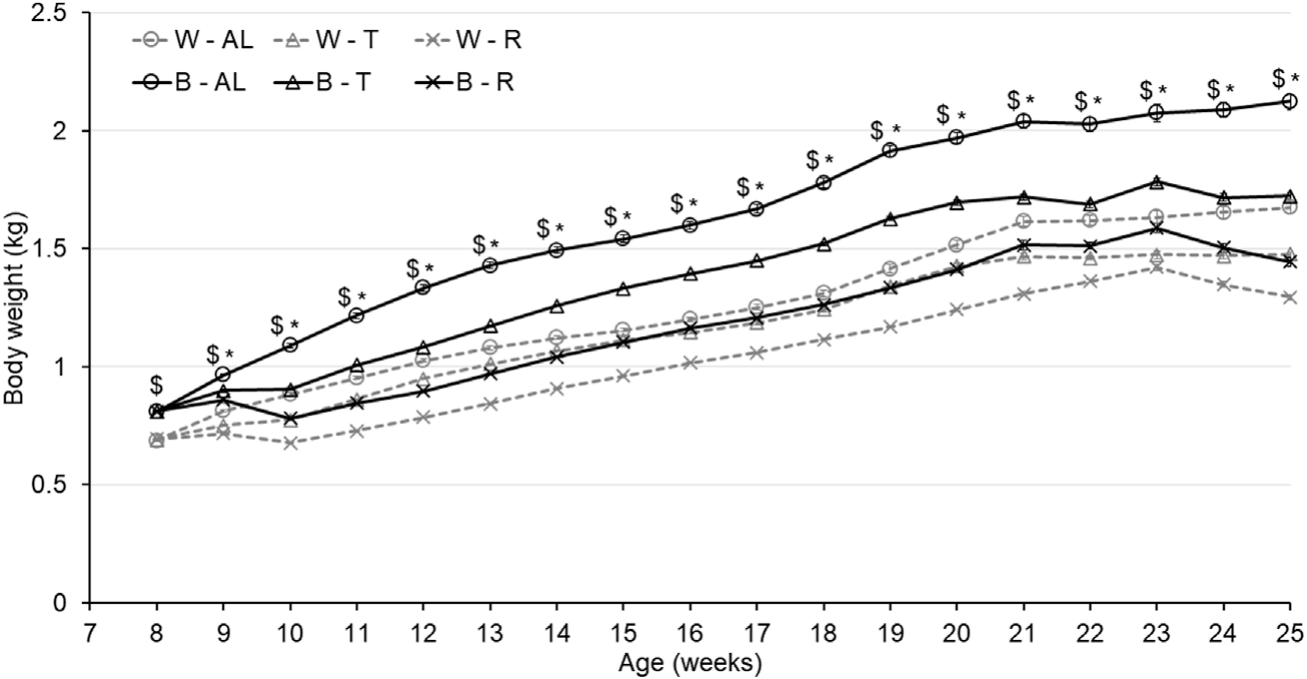
Weekly body weight of Lohmann LSL lite (W) birds and Lohmann Brown lite (B) birds reared under 3 feeding trajectories: *ad libitum* (AL), breeder’s recommended target (T) and Restricted (R). Values correspond to the mean ± SEM. *p*-values for the sources of variation: Age, *p* < 0.0001; Treatment, *p* < 0.0001; Strain, *p* < 0.0001; Age*Treatment, *p* < 0.0001; Age*Strain, *p* < 0.0001; Age*Strain*Treatment, *p* < 0.0001. The “$” symbols indicate significant differences between strain at specific time points while “*” indicates significant differences between treatment at specific time points.

As shown in Figure 1, the feeding strategy successfully generated 3 separate growth trajectories in both strains. The significant interaction between treatment and strain resulted in B birds displaying increased body weight gain compared to W birds from the beginning of treatment (8 woa) until the end of the trial (*p* < 0.0001). Furthermore, the significant interaction between age and treatment resulted in R birds displaying lower body weights regardless of strain (*p* < 0.0001) to achieve the expected 20% reduction in BW, while birds reared under AL feeding conditions were significantly heavier than birds reared under T (*p* < 0.0001). A three-way interaction between strain, treatment, and age was also observed (*p* < 0.0001) resulting in the weight of B-AL birds significantly higher than B-T from 10 to 25 woa (end of study). Interestingly, the BW of W-AL birds was initially higher than W-T from 10 to 12 woa, but this difference was no longer observed from 13 to 19 woa. However, starting at 20 woa, W-AL birds were significantly heavier than W-T and this difference remained throughout the end of the study. Ultimately, this resulted in AL birds being 400-g and 200-g heavier than T birds in the B and W strains, respectively, demonstrating a more prominent impact of treatment in B birds.

### 3.2 Reproduction

**FIGURE 2:**
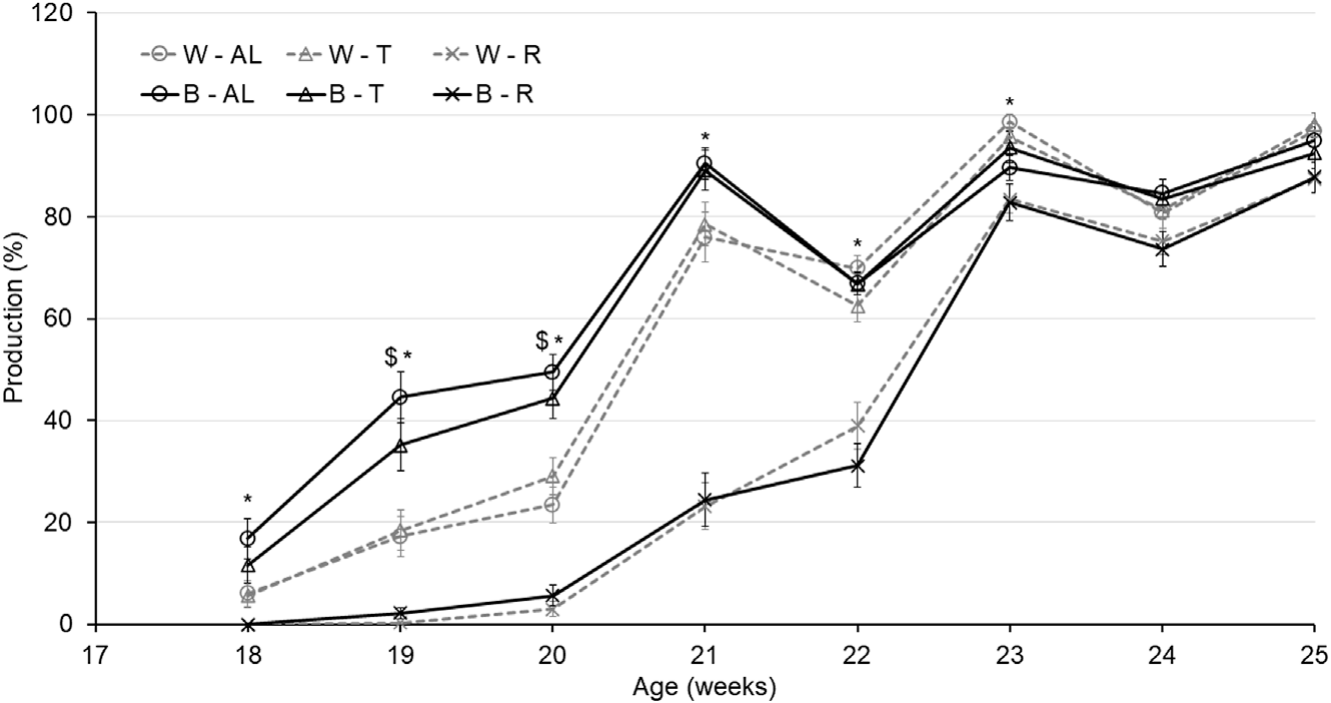
Egg production of Lohmann LSL lite (W) and Brown lite (B) birds reared under 3 feeding trajectories: *ad libitum* (AL), breeder’s recommended target (T) and Restricted (R). Data are expressed as egg/per housed hen on a weekly basis from 18 weeks of age to egg to 25 weeks of age. Values correspond to the means ± SEM. *p*-values for the sources of variation: Age, *p* < 0.0001; treatment, *p* < 0.0001; strain, *p* < 0.0001; Age*Treatment, *p* < 0.0001; Age*Strain, *p* < 0.0001; Age*Strain*Treatment, *p* < 0.05. The “$” symbols indicate significant differences between strain at specific time points while “*” indicates significant differences between treatment at specific time points.

**FIGURE 3:**
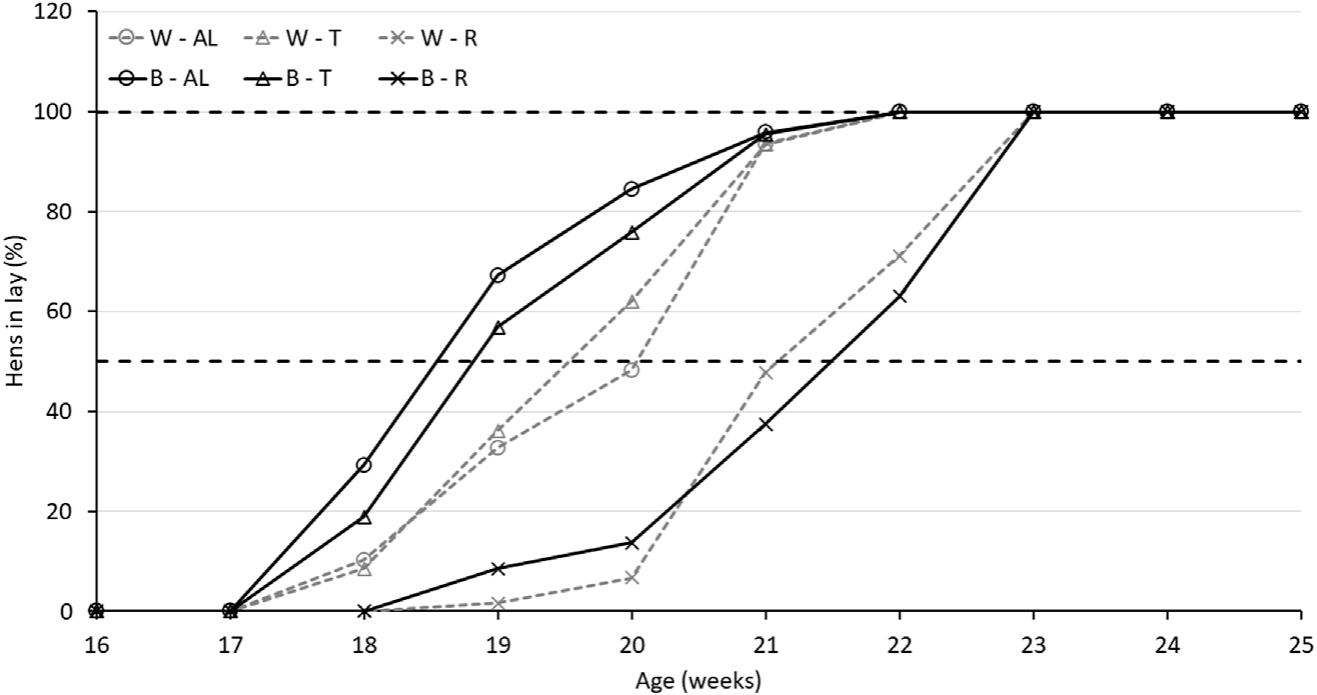
Percentage of Lohmann LSL lite (W) and Brown lite (B) birds reared under 3 feeding trajectories: *ad libitum* (AL), breeder’s recommended target (T) and Restricted (R) in lay.

Regarding AFE, there was an interaction between strain and treatment (*p* < 0.0001). While B-R birds were delayed by 16.7 days compared to B-AL, W-R birds were only delayed by 10.7 days compared to their AL counterparts. Interestingly, AFE for T birds did not differ from AL in either strain (Table 2). The aforementioned delay in AFE resulted in a significant interaction of strain, treatment, and age on egg production (*p* < 0.05). At 19 woa, B-AL hens had the highest production, with B-R and W-R hens demonstrating the lowest. However, by 20–22 woa, AL and T feeding resulted in higher production rates than R hens, regardless of strain (Figure 2). Despite lower production rates and a 2-week delay in AFE, 50% of R birds entered lay at around 21 woa which was delayed approximately by 1.5 weeks compared to W-AL and W-T, and 2.5 weeks compared to B-AL and B-T. However, all R hens entered lay by 23 woa, which was 1 week later than the AL and T hens (Figure 3). Furthermore, treatment and strain also affected BW at first egg (BW-FE; *p* < 0.0001), with B-AL birds displaying the highest BW-FE (2.00-kg) and W-R birds the smallest (1.40-kg; Table 2). However, there were no differences in BW-FE between B-R and W-AL.

**TABLE 2:** Age at first egg (AFE) and body weight at first egg (BW-FE) of Lohmann LSL lite (W) and Lohmann Brown lite (B) birds reared under 3 feeding trajectories: *ad libitum* (AL), breeder’s recommended target (T) and Restricted (R). Values correspond to the mean ± SEM. Different letters in different rows indicate significant differences between groups (*p* < 0.05).

		AFE	SEM	BW-FE	SEM
Strain	Treatment	days		g	
W	AL	142^b^	1.0	1590^c^	0.017
	T	140^b^	1.0	1480^d^	0.018
	R	152^a^	1.1	1400^e^	0.019
B	AL	135^c^	1.0	2000^a^	0.017
	T	138^bc^	1.0	1740^b^	0.017
	R	152^a^	1.1	1570^c^	0.019
**Source of variation**		***p*-value**			
Strain		<0.001		<0.001	
Treatment		<0.001		<0.001	
Strain*Treatment		<0.05		<0.001	

**FIGURE 4:**
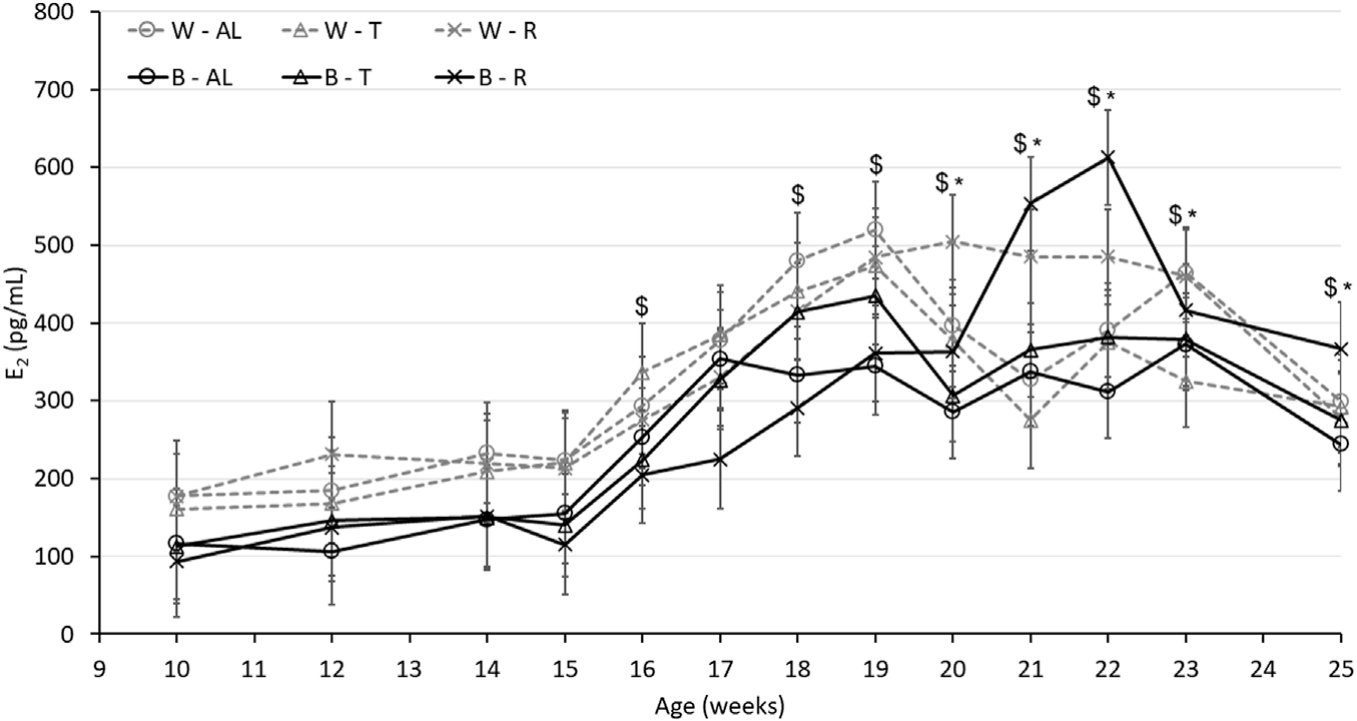
Plasma estradiol (E_2_) concentration of Lohmann LSL lite (W) and Brown lite (B) birds reared under 3 feeding trajectories: *ad libitum* (AL), breeder’s recommended target (T) and Restricted (R). Values are expressed as mean ± SEM of 10 birds/treatment/strain. *p*-values for the sources of variation: Age, *p* < 0.0001; treatment, *p* < 0.05; strain, *p* < 0.0001; Age*Treatment, *p* < 0.0001; Age*Strain, *p* < 0.0001; Age*Strain*Treatment, *p* < 0.0001. The “$” symbols indicate significant differences between strain at specific time points while “*” indicates significant differences between treatment at specific time points.

Plasma concentration of E_2_ was used as an indirect measure of the activation of the reproductive axis. In the absence of photostimulation, a single defined E_2_ peak was not observed among the focal birds (Figure 4). There was an interaction between strain, age, and treatment (*p* < 0.0001), with W-AL and W-R demonstrating higher E_2_ concentrations than B-AL and B-R, respectively, at 19 and 20 woa. Interestingly, at 21 and 22 woa regardless of strain, R birds had the highest E_2_ concentrations. Relative ovary weight, the number of SWFs and the number of LYFs further illustrate the differences in sexual maturation (Table 3). The interaction between age and treatment (*p* < 0.0001) indicates that relative ovary weights were higher in W birds starting from 20 woa until the end of the trial while B hens had heavier ovaries only at 20 and 22 woa. The significant interaction between age and treatment resulted in R birds showing the lowest number of LYFs regardless of strain (*p* < 0.0001) starting from 20 woa. The interaction between age and treatment also revealed that AL hens displayed a higher number of SWFs compared to R birds (*p* < 0.0001) starting from 22 woa and, the significant interaction between strain and age (*p* < 0.0001) resulted in more numerous SWFs in B hens at the beginning of maturation (18 woa). However, this difference dissipated between 20 and 22 woa. Interestingly, at 25 woa when all W birds were in lay, the number of SWFs was increased in W compared to B hens, suggesting a higher reproductive capacity (Table 3).

**TABLE 3:** Plasma estradiol concentrations, ovary weight, number of follicles, and body composition of Lohmann LSL lite (W) and Lohmann Brown lite (B) birds reared under 3 feeding trajectories: *ad libitum* (AL), breeder’s recommended target (T) and Restricted (R) at 14, 16, 18, 20, 22, and 25 weeks of age. Values correspond to the mean ± SEM (*n* = 6 birds/treatment/strain). NS, not significant; ND, not detected, E_2_, estradiol; LYFs, large yellow follicles; SWFs, small white follicles; BMC, bone mineral content; BMD, bone mineral density. Different letters indicate significant differences in the appropriate age.

	Follicles										Tissue				Bone			
Strain	Age (wk)	Treatment	E_2_	SEM	Ovary	SEM	LYFs	SEM	SWFs	SEM	Fat	SEM	Lean	SEM	BMC	SEM	BMD	SEM
			pg/mL		% BW						%		%		g		g/cm^2^	
W	14	AL	249	52.6	0.06	0.024	ND	—	ND	—	13.6	1.45	86.4	1.52	24.1	2.57	0.22	0.011
		T	176	52.5	0.06	0.024	ND	—	ND	—	11.7	1.45	88.3	1.52	25.8	2.57	0.23	0.011
		R	96	52.5	0.06	0.024	ND	—	ND	—	8.5	1.45	91.6	1.52	24.1	2.57	0.23	0.011
	16	AL	265	52.6	0.06	0.024	ND	—	ND	—	12.3	1.45	87.8	1.52	25.3	2.57	0.27	0.011
		T	258	52.5	0.06	0.024	ND	—	ND	—	9.7	1.45	90.3	1.52	27.4	2.57	0.28	0.011
		R	202	52.5	0.06	0.024	ND	—	ND	—	6.6	1.45	93.4	1.52	27.2	2.57	0.26	0.011
	18	AL	485	57.5	0.12	0.026	2.2	0.83	108	45.3	19.7	1.59	80.4	1.85	31.8	3.14	0.29	0.012
		T	460	52.6	0.11	0.024	1.7	0.76	100	42.0	16.6	1.45	83.5	1.52	35.4	2.57	0.31	0.011
		R	352	52.6	0.07	0.024	0.1	0.76	43	41.8	11.4	1.45	88.6	1.52	31.1	2.57	0.27	0.011
	20	AL	364	57.6	0.27^a^	0.024	6.3^a^	0.76	202	41.8	19.9	1.45	80.1	1.52	33.3	2.57	0.29	0.012
		T	393	52.5	0.25 ^ab^	0.024	5.1^a^	0.76	154	41.7	17.6	1.45	82.4	1.52	40.0	2.57	0.31	0.011
		R	461	52.5	0.13^b^	0.024	1.7^b^	0.76	117	41.7	15.9	1.45	84.1	1.52	36.2	2.57	0.29	0.011
	22	AL	449	52.6	0.38^a^	0.024	7.6^a^	0.76	456^a^	41.8	13.2	1.46	86.8	1.52	36.8	2.58	0.25	0.011
		T	401	52.6	0.39^a^	0.026	7.2 ^ab^	0.83	367 ^ab^	45.5	8.6	1.45	91.4	1.52	30.2	2.57	0.25	0.011
		R	499	52.6	0.21^b^	0.024	3.9^b^	0.76	211^b^	41.8	15.5	1.45	84.4	1.52	29.5	2.57	0.25	0.011
	25	AL	302	52.6	0.55^a^	0.024	7.0	0.76	729^a^	41.8	26.3	1.45	73.7	1.52	35.3	2.57	0.26	0.011
		T	251	52.6	0.52 ^ab^	0.024	6.4	0.76	559 ^ab^	42.0	20.7	1.45	79.3	1.52	32.3	2.57	0.28	0.011
		R	308	52.6	0.50^b^	0.024	5.6	0.76	378^b^	41.8	19.1	1.45	81.0	1.52	39.2	2.57	0.27	0.011
B	14	AL	142	52.6	0.06	0.024	ND	—	ND	—	18.4	1.45	81.6	1.52	29.2	2.57	0.25	0.011
		T	119	52.5	0.05	0.024	ND	—	ND	—	11.4	1.59	88.5	1.66	27.8	2.58	0.25	0.011
		R	77	52.6	0.05	0.024	ND	—	ND	—	11.1	1.59	88.9	1.66	27.2	2.57	0.23	0.011
	16	AL	237	57.5	0.07	0.024	ND	—	ND	—	17.3	1.45	82.7	1.52	33.2	2.57	0.28	0.012
		T	312	52.6	0.07	0.024	ND	—	ND	—	14.7	1.59	89.1	1.52	34.9	2.57	0.28	0.011
		R	114	52.6	0.05	0.024	ND	—	ND	—	9.2	1.59	90.7	1.66	34.5	2.57	0.28	0.011
	18	AL	490	52.5	0.16	0.024	0.7	0.76	204	41.7	17.4	1.45	82.6	1.52	38.3	2.57	0.31	0.011
		T	431	52.6	0.09	0.026	1.4	0.83	216	42.0	14.3	1.45	85.2	1.66	45.2	2.81	0.30	0.011
		R	269	52.6	0.06	0.024	0.0	0.76	134	42.0	10.6	3.53	89.4	3.70	32.1	2.57	0.26	0.011
	20	AL	330	52.6	0.27^a^	0.024	7.7^a^	0.76	290	41.8	18.3	1.45	81.7	1.52	38.9	2.57	0.28	0.011
		T	520	52.5	0.26^a^	0.024	7.0 ^ab^	0.76	297	41.7	12.8	1.45	87.3	1.52	42.8	2.57	0.30	0.011
		R	543	52.6	0.15^b^	0.024	3.8^b^	0.76	123	42.0	9.6	1.77	90.4	2.14	39.6	2.81	0.32	0.011
	22	AL	355	52.6	0.31^a^	0.024	8.2^a^	0.76	329	41.8	15.0	1.45	85.0	1.52	32.7	2.57	0.26	0.011
		T	430	52.6	0.33^a^	0.024	7.8^a^	0.76	256	41.8	16.0	1.45	84.0	1.52	35.0	2.57	0.28	0.011
		R	556	57.5	0.25^b^	0.026	3.4^b^	0.83	223	45.3	10.6	1.77	89.8	1.66	39.6	2.81	0.27	0.012
	25	AL	288	52.6	0.32	0.024	7.3	0.76	438	41.8	15.1	1.45	84.9	1.52	26.8^b^	2.57	0.28	0.011
		T	294	52.5	0.35	0.024	6.4	0.76	387	41.7	12.4	1.45	87.6	1.52	38.6 ^ab^	2.57	0.30	0.011
		R	387	52.6	0.31	0.024	5.7	0.76	303	42.0	11.1	1.59	88.9	1.66	43.1^a^	2.57	0.30	0.011

Source of variation			*p-value*															
Age			<0.001		<0.001		<0.001		<0.001		<0.001		<0.001		<0.001		<0.001	
Treatment			NS		<0.001		<0.001		<0.001		<0.001		<0.001		<0.05		<0.05	
Strain			NS		<0.001		NS		NS		<0.05		<0.05		<0.001		<0.01	
Age*Treatment			<0.001		<0.001		<0.001		NS		NS		NS		<0.01		NS	
Age*Strain			NS		<0.001		<0.05		<0.001		<0.001		<0.001		NS		NS	
Treatment*Strain			NS		NS		NS		NS		NS		NS		NS		NS	
Age*Treatment*Strain			NS		NS		NS		NS		<0.05		<0.05		NS		NS	

### 3.3 Body composition and correlations

Changes in BC including relative fat percentage, lean percentage, BMC and BMD are shown in Table3, 4. Treatment impacted the percentage of fat (*p* < 0.0001), with AL feeding increasing the overall relative fat deposition, while it was reduced for R and T (*p* < 0.0001), independent of strain (Table 3). Accordingly, AL feeding resulted in a lower lean tissue percentage compared to T and R (*p* < 0.0001; Table 3). Bone parameters were also impacted by the growth trajectories. The interaction between age and treatment indicates that BMD was higher (*p* < 0.05) in AL compared to R at 18 woa and was attenuated at 25 woa irrespective of the strains. R birds showed a lower BMC (*p* < 0.05) at 18 woa compared to T birds and had a higher BMC (*p* < 0.05) compared to AL at 25 woa. Furthermore, there was a moderate positive correlation between plasma E_2_ from the sampled birds and BMC (r = 0.467; *p* < 0.0001) and BMD (r = 0.415, *p* < 0.001) in R birds and a weak correlation in AL and T birds (Table 4). In addition, BMC had a strong (*p* < 0.0001) correlation with BMD irrespective of the treatment (Table 4).

**TABLE 4:** Correlation between plasma estradiol concentrations and bone parameters of Lohmann LSL lite (W) and Lohmann Brown lite (B) birds reared under 3 feeding trajectories: *ad libitum* (AL), breeder’s recommended target (T) and Restricted (R) before and after maturation. E_2_, estradiol; BMC, bone mineral content; BMD, bone mineral density. ^*^ *p* < 0.05;^**^ *p* < 0.001;^***^ *p* < 0.0001.

	*Ad Libitum*			Target			Restricted		
Trait	E_2_	BMC	BMD	E_2_	BMC	BMD	E_2_	BMC	BMD
**E_2_**	1	0.33*	0.366*	1	0.342**	0.321**	1	0.467***	0.415**
**BMC**		1	0.667***		1	0.745***		1	0.781***
**BMD**			1			1			1

## 4 Discussion

Beyond photostimulation, BW and BC play an important role in sexual maturation and egg production in chickens (Gous and Cherry, 2004; van der Klein et al., 2018b; van Emous et al., 2018; Zuidhof, 2018; Baxter and Bédécarrats, 2019; Hanlon et al., 2020; Hanlon et al., 2021). Therefore, this study aimed to investigate whether body growth and composition could impact spontaneous sexual maturation in brown and white strains of commercial layer hens. We hypothesized that differential growth trajectories would alter BC, and thus metabolic status. Specifically, a certain degree of body fat appears to be required to allow for sexual maturation to proceed in broiler breeders and quail (Bornstein et al., 1984; Yang et al., 2013). It was previously suggested that initiation of sexual maturation is governed not only by photoperiod (Chaney and Fuller, 1975) and age, but also by BW (Brody et al., 1980; Brody et al., 1984; Hanlon et al., 2021), body fat (Bornstein et al., 1984; Kwakkel et al., 1995), and lean body mass (Soller et al., 1984). As anticipated, R feeding reduced BW, while AL increased it, and this effect was more pronounced in B birds. This suggests that the recommended BW target in the management guidelines for B may not allow hens to reach satiety when housed individually, while guidelines for W do.

Changes in BW are positively associated with changes in BC (Heijmans et al., 2021). In line with this finding, our results from DXA indicate that the BC was strongly affected by the feeding regimens. Feed restriction resulted in the lowest percentage of body fat and this effect was more pronounced in B birds irrespective of the maturation status. It was reported that feed-restricted layers had a lower BW and abdominal fat pad compared to AL due to the distribution of nutrients available for maintenance, production, and storage (Murugesan and Persia, 2013). A 2.6-fold reduction in fat deposition was further observed in time-restricted laying hens due to an 11.7% reduction in feed intake and a 9.6% reduction in BW compared to control birds which had free access to feed throughout the light period (Saibaba et al., 2021). In addition, studies in broiler breeders revealed that relaxing feed restriction results in increased BW with a higher abdominal fat pad (Renema et al., 2001; Robinson et al., 2007; Van Emous et al., 2013; Van der Klein et al., 2018a; Salas et al., 2019; Heijmans et al., 2021). A relationship between BW and body fat mass during the rearing stage was established in broiler breeders (Sun and Coon, 2005; Van Emous et al., 2013; Salas et al., 2019), with BC positively correlated with feed allocation (Robinson et al., 2007). In the current study, the increase in fat content suggests that a body fat threshold between 10% to 15% is required to spontaneously initiate sexual maturation. The importance of such a fat threshold on sexual maturation was further studied in broiler breeders (van der Klein et al., 2018a; Zuidhof, 2018; Hadinia et al., 2020) and quail (Reddish et al., 2003). For instance, van der Klein et al. (2018a) reported that birds with a high BW had a higher fat pad proportion (2.2%) compared to the standard BW (1.6%), and the authors speculated that a fat pad mass threshold played a critical role on the process of sexual maturation. A similar concept was also proposed in quail (Zelenka et al., 1984; Yannakopoulos et al., 1995) and Yannakopoulos et al. (1995) specified that quail with lower body fat content displayed a delayed AFE, as they had not yet met the minimum body fat threshold. Lean tissue weight, representing protein levels in the body, was increased for AL and T treatments. However, rather than inducing sexual maturation, it may correspond to the development of reproductive organs, especially the oviduct. We further found that the ovary weight was higher in AL and T birds compared to R. Kwakkel et al. (1993) speculated that protein deposition in white leghorn pullets after 11 woa could be due to sexual maturity and reflect the development of the reproductive tract. In our case, total lean weight was higher in AL birds, but a concurrent elevation in fat deposition in this treatment resulted in an overall lower lean percentage compared to T and R hens. Eitan et al. (2014) emphasized the importance of the lean mass on sexual maturation in broiler breeders. The authors suggested that a body lean threshold is required to regulate sexual maturation rather than body fat content. However, our data suggest that rather than fat and lean content, the ratio (%) may be more important when a minimum BW has been achieved in layers.

Our results show that AL birds had a higher BW at first egg and displayed advanced maturations by 10.7 days in W and 16.7 days in B birds compared to those reared under R. Additionally, within B hens, AFE was further advanced by 2.9 days between T and AL. Interestingly, this corresponded to the observed increased growth trajectory and fat deposition for these birds under AL feeding. Surprisingly, the narrow 64 g BW threshold associated with sexual maturation reported by Hanlon et al. (2021) in white Leghorns derivatives, including the same W strain, was not observed in the current study. Though, in the present study, all W hens entered lay between 1.40 and 1.59 kg depending on treatment, which is comparable to the 1.43–1.49 kg BW range reported by Hanlon et al. (2021). Interestingly, the impact of treatment on BW-FE observed for B birds was much larger than for W suggesting that the possible BW threshold proposed by Hanlon et al. (2021) may depend on the strain (W *versus* B) and the growth trajectory. However, the impact of BC may be more consistent across strain and growth trajectory as we report that 10%–15% fat deposition is necessary for the onset of sexual maturations.

Beyond AFE, AL feeding was associated with improved development of reproductive organs with birds in the AL groups showing a higher ovary weight and the number of LYFs which resulted in higher egg productions irrespective of the strain. Furthermore, Time restricted feeding in laying hens resulted in lower BW, lighter egg weight, reduced egg production by 3.8% and persistency of egg laying by an average of 2.1% (Saibaba et al., 2021). In addition, previous studies in broiler breeders showed that high feed intake during the pubertal period elevates the number of hierarchal follicles (Hocking et al., 1987; Katanbaf et al., 1989; Yu et al., 1992) and accelerates the sexual maturation process (Wilson and Harms, 1986; Yu et al., 1992). Hocking (1993) reported that birds with a higher BW had more LYFs and a positive linear connection between the number of LYFs, BW and food intake was observed.

One key process during sexual maturation is the production of E_2_ by SWFs. In turn, E_2_ prepares the hen for active egg laying by switching the physiology and nutrient partitioning toward the synthesis of egg components (Hanlon et al., 2022) through the alteration of liver and bone physiology. During sexual maturation, E_2_ stimulates genes responsible for egg yolk and egg white production by binding to its receptor (ERα) in the liver (Bergink and Wallace, 1974; Kirchgessner et al., 1987; Flouriot et al., 1996) and transporting these components to the oviduct (Ratna et al., 2016). We report here that birds in the W group had an overall higher plasma E_2_ than those in B. However, the lack of photostimulation most likely resulted in a lack of synchronization within the treatment and a distinct identifiable peak in E_2_ could not be observed in the focal birds. Non-etheless, in the sampled birds, the increase in E_2_ concentration was delayed by a couple of weeks in R birds. The delay in egg production in R birds in the current study could be due to the delay in the production of E_2_ irrespective of the strains as it was reported in broiler breeders that birds receiving R feed during the rearing period had delayed E_2_ production followed by later AFE (Onagbesan et al., 2006), but higher cumulative egg production (van der Klein et al., 2018b). Unfortunately, this study only focused on the period leading up to peak production, and further studies should consider the impact on the remainder of the laying cycle.

In addition to lean and fat carcass content, the bone health of laying hens is critical to reproductive success. Due to the amount of Ca required for eggshell formation, high-producing strains of layer chickens are prone to Ca imbalance, which can result in osteoporosis near the end of the laying cycle (Webster, 2004). The onset of this disorder is said to be dependent on both laying performance and plasma E_2_ concentrations (Eusemann et al., 2022). The rise in plasma E_2_ at the initiation of the laying cycle shifts skeletal development from longitudinal growth to medullary formation, which results in calcium storage (Miller and Bowman, 1981; Dacke et al., 1993; Whitehead and Fleming, 2000) to support eggshell formation. Thus, exogenous administration of E_2_ in laying hens and roosters resulted in weaker bone strength compared to the untreated birds (Urist and Deutsch, 1960; Chen et al., 2014). E_2_ also elevates the levels of calcitriol, the active form of vitamin D, receptors in the intestine mucosa to enhance the uptake of dietary sources of Ca (for review: Hanlon et al., 2022). Hanlon et al. (2021), Hanlon et al. (2022) reported that the modern strains of layers exhibit recurrent elevations of E_2_ throughout the laying cycle, which may trigger the medullary bone formation to help provide an adequate Ca source during the laying period. They further suggested that the recurrent elevations of E_2_ positively correlated with medullary BMD and negatively correlated with cortical BMD.

However, although DXA provides an overall BMC and BMD value, our results demonstrated that B birds had higher BMC and BMD compared to W birds. BMC and BMD were lower at 18 woa and higher at 25 woa in R birds. Thus, the increased levels of BMC and BMD in both strains under R compared to AL birds could result from the elevations of plasma E_2_ concentrations at 22 woa. Furthermore, the results of the current study revealed a positive correlation between E_2_ and BMC and BMD which could be due to the development of medullary bone formation to support egg production as proposed by Hanlon et al. (2022), demonstrating a positive correlation between E_2_ and medullary BMC and BMD through a 100-week laying cycle of hens to provide enough amount of Ca for the support of egg production.

## 5 Conclusion

Feed allocation impacted growth and BC in a strain-dependent manner, resulting in differing sexual maturation and egg production. Specifically, higher BW, body fat percentage, and plasma E_2_ concentrations increased egg production and advanced AFE in birds which were reared under AL feeding conditions, which was more pronounced in B. Therefore, a body fat threshold between 10% to 15% appears to be required for the occurrence of spontaneously sexual maturation in laying hens. Furthermore, the positive correlation of plasma E_2_ with BMC and BMD highlights the importance of E_2_ and BC on sexual maturation in laying hens.

## Data Availability

The raw data supporting the conclusion of this article will be made available by the authors, without undue reservation.
